# Training and well-equipped facility increases the odds of skills of health professionals on helping babies breathe in public hospitals of Southern Ethiopia: cross-sectional study

**DOI:** 10.1186/s12913-019-4772-z

**Published:** 2019-12-09

**Authors:** Abera Mersha, Shitaye Shibiru, Teklemariam Gultie, Nega Degefa, Agegnehu Bante

**Affiliations:** 1 Department of Nursing, College of Medicine and Health Sciences, Arba Minch University, Arba Minch, Ethiopia; 2 Department of Midwifery, College of Medicine and Health Sciences, Arba Minch University, Arba Minch, Ethiopia

**Keywords:** Helping babies breathe, Neonatal resuscitation, Management of neonatal complications

## Abstract

**Background:**

Health professionals equipped with the adequate skills of helping baby breath remain the backbone in the health system in improving neonatal outcomes. However, there is a great controversy between studies to show the proximate factors of the skills of health care providers in helping babies breathe. In Ethiopia, there is a paucity of evidence on the current status of health care provider’s skills of helping babies breathe despite the improvement in neonatal health care services. Therefore, this study intends to fill those gaps in assessing the skills of helping babies breathe and its associated factors among health professionals in public hospitals in Southern Ethiopia.

**Methods:**

A facility-based cross-sectional study was conducted among 441 health professionals from March 10 to 30, 2019. A simple random sampling method was used to select the study participants. The data were collected through pre-tested interviewer-administered questionnaire and observational checklist. A binary logistic regression model was used to identify significant factors for the skills of helping babies breathe by using SPSS version 25. The *P*-value < 0.05 used to declare statistical significance.

**Results:**

Overall, 71.1% (95%CI: 66.2, 75.4%) of health professionals had good skills in helping babies breathe. Age group from 25 to 34 (AOR = 2.24; 95%CI: 1.04, 4.81), training on helping babies breathe (AOR = 2.69; 95%CI: 1.49, 4.87), well-equipped facility (AOR = 2.15; 95%CI: 1.09, 4.25), and adequate knowledge on helping babies breathe (AOR = 2.21; 95%CI: 1.25, 3.89) were significantly associated with a health professionals good skill on helping babies breathe.

**Conclusions:**

Even though a significant number of care providers had good skills in helping babies breathe, yet there is a need to further improve the skills of the provider in helping babies breathe. Hence, health facilities should be equipped with adequate materials and facilitate frequent training to the provider.

## Background

Achieving the Sustainable Development Goal (SDG) targets for ending preventable mortality and the provision of universal health coverage will require large-scale approaches to improving the quality of health care [1]. Globally, significant improvement has been registered in reducing under-five mortality. However, a reduction in neonatal death remains stagnant particularly in sub-Saharan Africa [2, 3].

Neonatal mortality accounts for an increasing proportion of mortality in under-five children worldwide. Most newborn deaths befall in low and middle-income countries. The majority of neonatal mortality are found in sub-Saharan Africa [4, 5]. Birth asphyxia causes approximately 27 to 30% of neonatal deaths in resource-limited countries [6]. By providing quality care during delivery and the postpartum period, around 2/3 of the world’s neonatal mortality may be prohibited [7, 8].

Countrywide, great achievement has been marked by the application of helping babies breathe (HBB) [8]. After delivery, newborns who are breathing and crying may have waited at least for one to three minutes for cord clamping, but for those who are not, a cord is clamped immediately and resuscitation initiated [9]. Helping babies breathe is a set of interventions used to assist the airway, breathing, and circulation (ABC of life) of a newborn following the birth or to help it breathe and to help its heart beat [10, 11]. Infants who required face mask ventilation (FMV) were more likely to die predominantly when the intervention was not given on time or sustained [12].

Health care providers are responsible for HBB. During resuscitation majority of newborns respond with simple steps, such as drying, stimulation, warmth, and bag-mask ventilation [13, 14]. Effective application of neonatal resuscitation for those in need is identified squandered occasions to provide lifesaving care and highlighted the need to improve the accessibility of indispensable apparatus and ensure that birth attendants acquire and maintain their skills to provide high-quality newborn care [15].

Accessibility and utility of different apparatus, and having skillful personnel is very vital to comprehend the advantage from HBB [7, 8]. Good skill of health professionals on the HBB is essential for improving the neonatal outcome. It has an inordinate upshot to identify, manage, and avoid unwanted outcomes [16, 17]. Based on the evidence, health professionals have the supremacy to improve the excellence of care in the health care delivery system [18].

The success of HBB depends on the provider’s clinical skill as well as how to perform essential steps and how to access basic HBB equipment. Report from studies done in Africa and Asian countries showed that well-trained health professionals on the HBB are not steadily available in all health care facilities [19, 20]. Studies conducted in Lake Zone and 12 regions of Tanzania stated that health care provider’s skills about helping babies breathe were 32.4, and 0.03% respectively [21, 22]. Similarly, a study in Cameron showed health care providers were competent at providing essential newborn care, but they lacked skills for proper handling of newborns that do not breathe at birth, only 24% of tasks [23]. In Nigeria, 97% of delivery attendants do not execute the competencies on resuscitation [15]. The overall skills of midwives, nurses, pediatrics residents, and OB-GYN residents were insufficient as shown in a study done in Ethiopia, the overall mean was 6.8 (SD = 3.9) [24].

Lack of professional support and infrequent resuscitation skills practice are commonly cited as barriers to skill retention after HBB training [25]. The working facility, experience, limited provider knowledge, inadequate training, and poor availability of equipment were determinants of health professional skill in newborn resuscitation [13, 22, 23, 26, 27].

Skills learned in standardized courses are estimated to last only a few months. Neonatal Resuscitation Program skills or HBB deteriorates immediately after certification. Significant skill deficits were seen at baseline raising concerns regarding the efficacy of the current course structure. Discrepancies in knowledge and skill retention may impact caregiver performance [28].

There is a great controversy between studies to show the significant factors that affect the skills of health care providers. Some studies did not assess the most proximal factors of skills on HBB like facility-related factors. But other studies revealed a trivial association and does not assess the skills of health care providers as well as their association. Besides, there is a scantiness of information’s in Ethiopia that shows the skills about HBB, despite the improvement in neonatal health care services to the knowledge of the investigator. Therefore, this study fills those gaps by assessing the current skills of HBB and factors affecting in the hospitals of Gamo, Gofa, Segen Areas People, Konso and South Omo Zone, Southern Ethiopia.

## Methods

### Study setting and period

This study was conducted in the Hospitals of Gamo, Gofa, Segen Areas People, Konso and South Omo Zone, Ethiopia from March 10–30, 2019. Gamo, Gofa, Segen Areas People, Konso and South Omo area are administrative Zones in the Southern part of Ethiopia. Those Zones hosted different general and primary hospitals, which serve the community by providing preventive and curative services. There are five functional hospitals in the Gamo Zone (Arba Minch General Hospital, Chencha Primary Hospital, Kamba Primary Hospital, Gerese Primary Hospital and Selamber Primary Hospital), one hospital in Gofa Zone (Sawla General Hospital), two hospitals in Segen Areas People Zone (Gidole Primary Hospital and Amaro Kele Primary Hospital), one hospital in Konso Zone (Karat Primary Hospital) and two hospitals in South Omo Zone (Jinka General Hospital and Gazer Primary Hospital).

### Study design

The facility-based cross-sectional study design was employed.

### Population

#### Source population

The source population was all health professionals who were working in Hospitals of Gamo, Gofa, Segen Areas People, Konso and South Omo Zone, Southern Ethiopia.

#### Study population

The study population was all health professionals who were working on delivery, neonatal intensive care unit (NICU), pediatric wards and operating room (OR) in selected Hospitals.

#### Inclusion and exclusion criteria

All health professionals who were staff in the respective wards in each hospital were included in this study, whereas those health professionals on annual leave at the time of data collection were excluded from this study.

#### Sample size determination

The sample sizes for all objectives were computed using Epi info 7 stat Calc. For the first specific objective (to assess the skills on helping babies breathe) single population proportion was used by considering the following assumption: *P* = 0.475 from the study conducted in Ethiopia [24], 95% level of confidence and 5% margin of error. To determine the sample size for the 2nd objective (to identify associated factors) two-sample comparison proportions were used. The sample size of the 1st objective was greater than that of the 2nd objective. So, a non-response rate of 15% was added to the sample size of the 1st objective (*n* = 383) to come up with the final sample size. As a result, the calculated sample size for this study was 441.

#### Sampling techniques

**Fig. 1:**
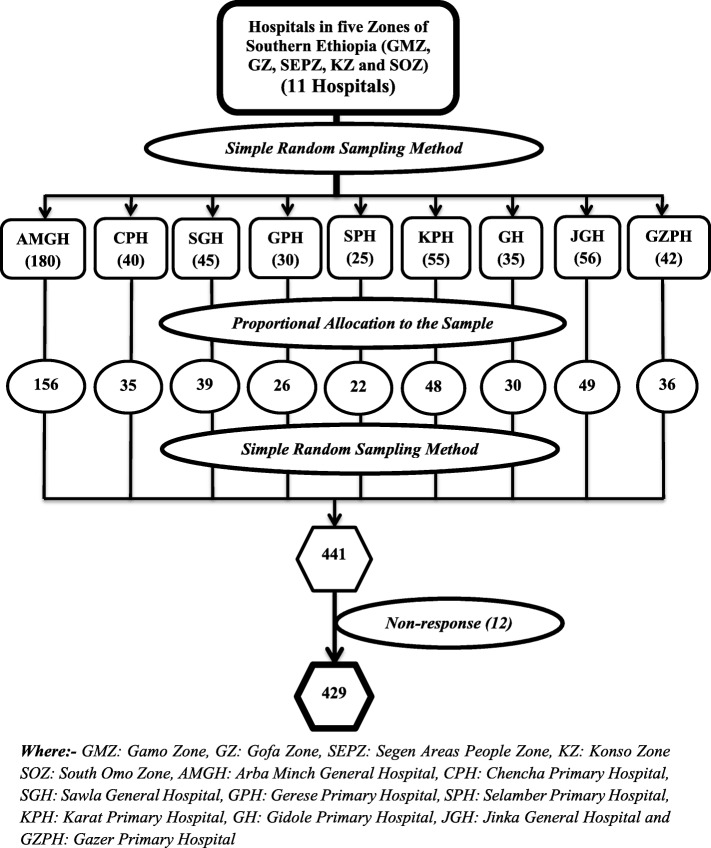
Schematic presentation of the sampling procedure for the study conducted among health professionals in public hospitals of Southern Ethiopia, 2019

There are eleven fully functional hospitals in five Zones of Southern Ethiopia (Gamo, Gofa, Segen Areas People, Konso and South Omo Zone). A simple random sampling method was used to select nine hospitals from them. Initially, the calculated sample size was proportionally allocated to each hospital based on the number of health professionals who were working on the respective wards of each hospital. Secondly, a table of the random number was used to select each health professionals based on proportions to come up with the calculated sample size (Fig. 1).

#### Data collection tools

A pre-tested structured both self and interview administered questionnaires and observational checklist were used to collect the data. Some tools were developed or adapted from different works of literature and guidelines. But, to measure the outcome variables, a standard questionnaire was used which were developed, reviewed, revised and approved in different workshops [22, 29–32]. The tool contains five main parts: basic characteristics, provider related questions, facility assessment tools (observational checklist and interview), knowledge assessment tools and skill assessment tools. The pre-test was carried out with 5% of study subjects in other health care institutions and necessary modifications and amendments were taken accordingly before actual data collection (Additional files 1 and 2).

#### Data collectors and data collection procedures

A well-trained three BSc holder nurses and six midwives for data collection, and three MSc holder nurse for supervision were involved. The data were collected from health professionals in nine selected hospitals, which were found in five zones of Southern Ethiopia. The data collectors were given information about the study aim and the possible procedures for study participants before providing the questionnaire and interviewing the study participants. After stating the study aim, the data collectors offered a self-administered questionnaire to assess the knowledge of helping babies breathe. Then, each study, participants were interviewed for the skills of helping babies breathe in the room which was arranged by data collectors during an off-work time after returning the self-administered questionnaire. In the end, key informants were interviewed and observational checklists were used to assess the hospitals for the availability of guidelines, essential items, and infrastructures.

#### Study variables

Health professional’s skills of HBB were the dependent variable, and socio-demographic characteristics, provider-related factors, facility-related factors, and knowledge on HBB were independent variables for this study.

#### Measurements

The description and measurements of the outcome and some explanatory variables used in this study were stated in detail below (Table 1).

**Table 1:** Operational definitions of variables and measurements to study conducted among health professionals in public hospitals of Southern Ethiopia, 2019

Variables	Descriptions
Health professionals	Health care providers (nurses, midwives, health officers, Integrated Emergency Surgery and Obstetrics (IESO) and medical doctors) who were working in the delivery ward, NICU, pediatric ward and OR and have access to HBB.
Knowledge of health professionals on HBB	Level of awareness of the health care providers on HBB. Knowledge of health professionals was considered adequate when they answered correctly at least 16 out of 20 knowledge, assessing questions on helping babies breathe (total score of ≥80%) after following algorithms for the main American Heart Association (AHA) advanced cardiac life support, and inadequate when they correctly answered less than 16 out of 20 questions (total score < 80%) [32, 43].
Skills of health professionals on HBB	Skills of health professionals were good for who responded correctly at least 32 of the 40 activities on skill assessment tools for HBB (total score of ≥80%) after following algorithms for the main AHA advanced cardiac life support, and poor for who correctly responded in less than 32 of the 40 activities (total score < 80%) [24, 31].
Well-equipped facility	Those facilities that have both essential items (mucus extractors, infant Ambu bag, face masks, towels, and newborn resuscitation table) and priority items (syringes, suction apparatus, stethoscope for use with newborns and source of warmth).

#### Data quality control

To maintain the validity of data to be collected randomly selected study participants were involved and standard questionnaires and checklist were used. The pre-test was conducted for inconsistencies and misunderstandings of the questions to assure reliability. Extensive training was given to data collectors and supervisors regarding the objective of the study, data collection tool, ways of data collection, checking the completeness of data collection tools and how to maintain confidentiality to maintain the quality of data to be collected. All data were checked for completeness, accuracy, clarity, and consistency by the principal investigator and by supervisors, on the day of data were collected. Data were checked for completeness before data entry into the software. Proper coding and categorization of data were maintained in the quality of the data to be analyzed.

#### Data processing and management

The data collectors, supervisors, and the corresponding author were in charge of verifying the fullness of the information. The data collectors filled in the date and signed each questionnaire, which was well ahead plaid, corrected and signed by the supervisors regularly. Hard copies were used to collect the data and kept in a locked cabinet by each supervisor until gathered by the principal investigator during supervision. Then, the data were coded, cleaned, edited and entered into Epi data version 3.1 to minimize logical errors and design skipping patterns, and exported to SPSS version 25 for analysis.

#### Data analysis

Univariate, bivariate, and multivariable analysis was done. The assumptions for binary logistic regression were checked. Hosmer-Lemeshow statistic and Omnibus tests were done for model fitness. Variables with *P* < 0.25 in the bivariate analysis, variables that were significant in previous studies, and context point of view were considered to select the candidate variables for the final model. Collinearity statistics (Variance inflation factor (VIF) > 10 and tolerance (T) < 0.1 were considered as suggestive of the existence of multi co-linearity. Adjusted Odds Ratio along with 95% CI was estimated to identify factors affecting health professional’s skills of HBB. The *P*-value < 0.05 was considered to declare a result as statistically significant. Then simple frequencies, summary measures, tables, and figures were used to present the information.

## Results

### Socio-demographic characteristics of the respondents

In this study, 429 health professionals participated, yielding a response rate of 97.3%. The mean age and standard deviation of study participants were 29.7 ± SD4.88 years old. More than half (55.9%) of the study participants were females and 281(65.5%) were married. Two hundred (46.6%) of health professionals were orthodox religion followers (Table 2).

**Table 2:** Socio-demographic characteristics of the health professionals in hospitals of Southern Ethiopia, 2019

Characteristics	Frequency	Percentage (%)
Age of respondent		
15–24	48	11.2
25–34	317	73.9
≥ 35	64	14.9
Sex		
Male	189	44.1
Female	240	55.9
Marital status		
Married	281	65.5
Divorced	17	4
Widowed	4	0.9
Single	127	29.6
Religion		
Orthodox	200	46.6
Protestant	207	48.3
Catholic	6	1.4
Muslim	16	3.7
Salary		
< 3579ETB	68	15.9
3579-5452ETB	254	59.2
> 5452ETB	107	24.9

Note: *1ETB = 0.036USD*

### Provider related factors

Of the total respondents, 248(57.8%) were nurses and 213(49.7%) were diploma holders. Two hundred forty-three (56.6%) of health professionals were working in general hospitals. One hundred sixty-seven (38.9%) of the health professionals had work experience of 3 to 6 years and almost half (52.0%) had been working for 12 to 52 months in the specified wards. Out of the total study participants, 333(77.6%) received training on HBB and 375(87.4%) had recent involvement in the help to baby’s breath. Regarding the type of training, 135(40.6%), 117(35.1%) and 81(24.3%) received pre, in and both pre and in-service respectively (Table 3).

**Table 3:** Provider related factors of the health professionals in hospitals of Southern Ethiopia, 2019

Variables	Frequency	Percentage (%)
Profession		
Nurse	248	57.8
Midwives	117	27.3
Health Officers	28	6.5
IESO	28	6.5
Medical Doctors	8	1.9
Qualification		
Diploma	213	49.7
BSc	173	40.3
MSc	35	8.2
General Practitioner	6	1.4
Specialists**®**	2	0.5
Unit of service		
Delivery	146	34.0
NICU	92	21.4
Pediatric Ward	130	30.3
OR	61	14.2
Year of experience in specified ward		
< 12 month	112	26.1
12–52 month	223	52.0
> 52 month	94	21.0
Year of experience in work		
< 3 year	172	40.1
3–6 year	167	38.9
> 6 year	90	21.0
Training on HBB		
Yes	333	77.6
No	96	22.4
Recent involvement in HBB		
Yes	375	87.4
No	54	12.6

®obstetrician and gynecologist, pediatrician

### Facility related factors

The number of deliveries occurred in facilities of Gamo, Gofa, Segen Areas People, Konso and South Omo Zone in the past 12 months was ranged from 487 to 4640 and on average 1488 deliveries. On the other hand, the number of newborn deaths occurred within 24 h after delivering in the past 12 months was ranging from 3 to 46 and 11 deaths on average. All the facilities were assessed for the performance of newborn resuscitation and all had performed with the minimum of one newborn’s resuscitation in the past 3 months. Besides, facilities were categorized as a primary and general hospital and assessed for the availability of guidelines, essential items, priority items, and infrastructures (Table 4).

**Table 4:** Facility related factors for the study conducted among health professionals in public hospitals of Southern Ethiopia, 2019

Facility	Primary Hospitals (*n* = 6)		General Hospitals (*n* = 3)	
	Frequency	(%)	Frequency	(%)
Availability of guidelines				
Management of newborn complications	6	100	3	100
Postnatal care of newborns	4	66.7	2	66.7
Immediate newborn care	6	100	2	66.7
Availability of essential items				
Mucus extractor	6	100	2	66.7
Infant Ambu bag	6	100	2	66.7
Infant face masks (sizes 0,1,2)	6	100	3	100
Towels or cloth for newborn	6	100	2	66.7
Newborn resuscitation table	6	100	3	100
Availability of priority items				
Syringes (1 ml, 2 ml, 5 ml, 10 ml)	6	100	2	66.7
Suction apparatus	6	100	3	100
Stethoscope for use with newborns	4	66.7	3	100
Source of warmth	6	100	3	100
Infrastructures^a^(mean ± SD)	50 (1.17)		33.3 (1.53)	

^a^electricity, generator, availability of water in different parts of the facility, various kinds of telephone, radio, television, light source, ventilation, toilet, heating, fan or air conditioning, curtains for patient privacy and waiting

### Knowledge of helping babies breathe

Regarding diagnosis of birth asphyxia, 390(90.9%) stated depressed breathing and 356(83.0%) indicated initial steps of newborn resuscitation were positioning the head to slightly extended neck. Four hundred fifteen (96.7%) reported a place mask to cover chin, mouth, and nose while resuscitating with a bag and mask or tube and mask. Of the total respondents, 384(89.5%) stated initiate breastfeeding after 30 s if the baby is breathing and there is no sign of respiratory difficulty. The majority (90.9%) of the study participants stated that administer oxygen, if available and 370(86.2%) reported continue to ventilate if the newborn does not initiate breathing, or breathing is less than 30 per minute, or if there is an intercostal retraction or grunting (Table 5).

**Table 5:** Knowledge of helping babies breathe among health professionals in public hospitals of Southern Ethiopia, 2019

Categories	Frequency	Percentage (%)
How do you diagnose birth asphyxia?		
Depressed breathing	390	90.9
HR < 100/min	354	82.5
Central cyanosis	340	79.3
What are the initial steps of newborn resuscitation?		
Place newborn face up	343	80.0
Wrap or cover baby	355	82.8
Position head so the neck is slightly extended	356	83.0
Aspirate mouths and then nose	342	79.7
Explain to mother what is happing	344	80.2
What do you do when resuscitating with a bag and mask or tube and mask?		
Place mask to cover chin, mouth, and nose	415	96.7
Ensure seal between mask and face	387	90.2
Ventilate 1 or 2 times and see if the chest is rising	386	90.0
Ventilate 40 times per minute for 1 min	359	83.7
Pause to determine whether the baby is breathing spontaneously	356	83.0
What do you do if the baby is breathing and there is no sign of respiratory difficulty? After 30 s		
Keep baby warm	383	89.3
Initiate breastfeeding	384	89.5
Continue monitoring the baby	379	88.3
What do you do if the baby does not begin breathing, breathing is less than 30 per minute, or if there is intercostal retraction or grunting?		
Continue to ventilate	370	86.2
Administer oxygen, if available	390	90.9
Assess the need for special care	350	81.6
Explain to mother what is happening	350	81.6

Overall, 76.2% (95%CI: 72.2, 80.3%) of the study participants had adequate knowledge in helping babies breathe.

### Skills of helping babies breathe

Of the study participants, 347(80.9%) itemized make sure equipment is ready for use before staring resuscitating with bag and mask and 404(94.2%) stated position to head in a slightly extended position while resuscitating by using a bag and mask. During ventilation, three hundred forty-eight (81.1%) stated that observe chest for easy rise and fall if the newborn chest is not rising 376(87.6%) reported that reposition mask to improve sealing. After resuscitation, if breathing is normal (no indrawing or grunting) 386(90.0%) of the study participants stated the place the baby in skin-to-skin contact with the mother and observe breathing at frequent intervals. The majority (90.0%) stated that if newborn breathes with severe chest indrawing, ventilate with oxygen if available. Three hundred fifty-five (82.8%) stated that disinfect mucous extractors with chemicals, and rinse all parts with clean water and allow to air dry for reusable (Table 6).

**Table 6:** Skills of helping babies breathe among health professionals in public hospitals of Southern Ethiopia, 2019

Categories	Frequency	Percentage (%)
Getting ready		
Make sure equipment is ready for use	347	80.9
Wash hands and wear gloves	343	80.0
Quickly dry and wrap or cover the newborn	314	73.2
Place newborn on the back on the clean, warm surface	288	67.1
Tell women what is going to be done, listen to her, and respond to her questions and concerns	282	65.7
Provide emotional support and reassurance	282	65.7
Resuscitating using a bag and mask		
Position head in the slightly extended position	404	94.2
Suction first the mouth and then the nose	392	91.4
Introduce catheter into mouth and suction	372	86.7
Introduce catheter into each nostril and suction	376	87.6
Suction well if blood or meconium is on the newborn’s mouth and/or nose	383	89.3
If the baby is still not breathing, start ventilating	390	90.9
Recheck position of newborn’s head	373	86.9
Place the correct-sized mask on the newborn’s face	367	85.5
Form a seal between the mask and the newborn’s face	350	81.6
Squeeze bag	338	78.8
Check the seal by ventilating and observing chest rise	353	82.3
If the newborn’s chest is rising:		
Ventilate at 40 breaths/minute	344	80.2
Observe chest for easy rise and fall	348	81.1
If the newborn’s chest is not rising:		
Check the position of the head again	367	85.5
Reposition mask to improve the seal	376	87.6
Squeeze the bag harder; repeat suction	361	84.1
Ventilate for 1 min and then assess if the newborn is breathing	363	84.6
If breathing is normal (no indrawing or grunting):		
Place in skin-to-skin contact with mother	386	90.0
Observe breathing at frequent intervals	386	90.0
Encourage the mother to begin breastfeeding	378	88.1
If the newborn is breathing with severe indrawing:		
Ventilate with oxygen, if available	386	90.0
Arrange immediate transfer for special care	354	82.5
If there is no gasping or breathing at all after 20 min of ventilation, stop ventilating	352	82.1
Post-procedure tasks		
Place disposable suction catheters and mucus extractors in a leak-proof container	354	82.5
For reusable catheters and mucus extractors		
Place in a chlorine solution for 10 min	354	82.5
Wash in water and detergent	354	82.5
Use a syringe to flush catheters/tubing	351	81.8
Boil or disinfect in a chemical solution	334	77.9
Take apart valve/mask and inspect for cracks/tears	333	77.6
Wash valve/mask and check for damage	339	79.0
Select sterilization or high-level disinfection method	346	80.7
Wash hands and dry with a clean cloth or air entry	350	81.6
After chemical disinfection, rinse all parts with clean water and allow to air dry	355	82.8

Overall, 71.1% (95%CI: 66.8, 75.4%) of the study participants had good skills on helping babies breathe.

### Factors associated with the skills of helping babies breathe

Age, training on HBB, well-equipped facility, and adequate knowledge on HBB had significantly associated with a health professional’s skills of HBB after controlling for confounders in the multivariable model.

Health professionals whose ages ranged from 25 to 34 years old were 2.24 times more likely had skills on HBB as compared to the age group from 15 to 24 years old (AOR = 2.24,95%CI:1.04, 4.81). Those who received training on HBB were 69% more likely had skills on HBB (AOR = 2.69, 95%CI: 1.49, 4.87). The odds of skills of HBB among health professionals who working in a well-equipped facility was 2.15 times (AOR = 2.15, 95%CI: 1.09, 4.25). Those health care providers who had adequately knowledgeable on HBB were 2.21 times more likely had skills on HBB (AOR = 2.21, 95%CI: 1.25, 3.89) (Table 7).

**Table 7:** Factors associated with the skills of helping babies breathe among health professionals in public hospitals of Southern Ethiopia, 2019

Variables	Skills of HBB		Crude OR	Adjusted OR
	Good	Poor	95%CI	
Age				
15–24	23 (47.9%)	25 (52.1%)	1	1
25–34	236 (74.4%)	81 (25.6%)	3.17 (1.70,5.89)	2.24 (1.04,4.81)*
> 34	46 (71.9%)	18 (28.1%)	2.78 (1.27,6.09)	1.92 (0.68,5.39)
Salary				
< 3579ETB	42 (61.8%)	26 (38.2%)	1	1
3579-5452ETB	174 (68.5%)	80 (31.5%)	1.35 (0.77,2.35)	0.90 (0.43,1.89)
> 5452ETB	89 (83.2%)	18 (16.8%)	3.06 (1.51,6.19)	1.13 (0.38,3.42)
Profession				
Nurse	167 (67.3%)	81 (32.7%)	0.64 (0.42,0.99)	0.72 (0.43,1.22)
Other**©**	138 (76.2%)	43 (23.8%)	1	1
Qualification				
Diploma	133 (62.4%)	80 (37.6%)	0.43 (0.28,0.66)	0.55 (0.29,1.03)
Other**®**	172 (79.6%)	44 (20.4%)	1	1
Year of experience in specified ward				
< 12 month	60 (53.6%)	52 (46.4%)	1	1
12–52 month	169 (75.8%)	54 (24.2%)	2.71 (1.68,4.39)	1.61 (0.90,2.86)
> 52 month	76 (80.9%)	18 (19.1%)	3.66 (1.94,6.89)	1.83 (0.83,4.03)
Year of experience in work				
< 3 year	116 (67.4%)	56 (32.6%)	1	1
3–6 year	120 (71.9%)	47 (28.1%)	1.23 (0.78,1.96)	0.99 (0.54,1.82)
> 6 year	69 (76.7%)	21 (23.3%)	1.59 (0.89,2.84)	0.90 (0.41,1.97)
Training on HBB				
Yes	258 (77.5%)	75 (22.5%)	3.59 (2.23,5.77)	2.69 (1.49,4.87)*
No	47 (49.0%)	49 (51.0%)	1	1
A recent performance on offering HBB				
Yes	281 (74.9%)	94 (25.1%)	3.74 (2.08,6.71)	1.78 (0.82,3.89)
No	24 (44.4%)	30 (55.6%)	1	1
Confidence in offering HBB				
Very confident	237 (74.8%)	80 (25.2%)	3.56 (1.71,7.38)	1.68 (0.67,4.23)
Somewhat confide.	53 (67.1%)	26 (32.9%	2.45 (1.07,5.61)	1.99 (0.72,5.50)
Not confident	15 (45.5%)	18 (54.5%)	1	1
Well-equipped facility				
Yes	277 (75.3%)	91 (24.7%)	3.59 (2.06,6.26)	2.15 (1.09,4.25)*
No	28 (45.9%)	33 (54.1%)	1	1
Knowledge on HBB				
Inadequate	47 (46.1%)	55 (53.9%)	1	1
Adequate	258 (78.9%)	69 (21.1%)	4.38 (2.73,7.01)	2.21(1.25,3.89)*

©Midwives, Health officers, IESO and medical doctors,® BSc, MSc, GP and Specialists and *Significant at *P* < 0.05

## Discussion

The skills of health professionals on HBB is very pivotal to save the life of many neonates during the intrapartum period and to reduce neonatal mortality at large. But, conclusive evidence was lacked to show a clear direction for intervention in many settings including our country Ethiopia. The erstwhile studies were not assessed determinates for the skills of health professionals on HBB in a comprehensive way. Therefore, this study showed the recent status of health professionals on the skills of HBB and factors affecting in the study setting.

In this study, 71.1% (95%CI: 66.8, 75.4%) of the study participants had good skills in HBB. Age, training on HBB, well-equipped facility, and knowledge on HBB had significantly associated with health professionals skills on HBB.

The magnitude of good skills of health professionals on HBB was in line with a study done in Afghanistan (66 and 71%) and higher than studies done in Tanzania (32.4%), Iraq (52%) and Ethiopia (55.8%) [22, 24, 31, 33]. The reason for this discrepancy in the skills of HBB is because there is an advance in the health care system and refreshment pieces of training are given to health professionals by concerning bodies.

Age of the respondents ranged from 25 to 34 years old were significantly associated with the skills of HBB. But, this was incongruent with the studies done in Ethiopia [24, 32]. This is difference may be due methodological, those health professionals whose ages were in this category were received training on HBB, and the majority of health professionals are in this age group as from normal demography. Finding from this study showed that training on HBB, and working in well-equipped facilities were significantly associated with health professional’s skills on HBB. This is in line with different studies done Kenya, Afghanistan, Rwanda, Tanzania, India, developing countries, Ethiopia, Nepal and Global Network research sites (Nagpur and Belgaum, India and Eldoret, Kenya), low income countries, resource-limited settings, Cameroon and Dominican Republic [4, 13, 17, 22–24, 26, 32, 34–40]. The reason this is due to the fact health professionals who received training as well as refreshment training has recent memory and actively performs the activities. Similarly, those providers who are working in well-equipped facilities are frequent exposure to cases and equipment used for HBB and developing their skills from time to time.

As revealed in the study knowledge of health professionals on HBB was significantly associated with the skills of HBB. This is consistent with studies conducted in Kenya, Cameroon, Nepal, and Ethiopia [13, 23, 26, 27]. The fact for this is knowledgeable health care professionals are more likely having the skills and perform the activities in a good manner as much as possible.

In this study profession, and years of experience working in specified wards (NICU, and maternity unit) had no significant association with the skills of HBB. This is contradicted with the studies done in Tanzania, Kenya, and Afghanistan [21, 22, 31, 40, 41]. This is due to differences in the health care system (having the well-equipped facility, competent staff, advanced system, and quality of delivery) and methodological facets. Salary, academic qualification, year of experience in work, recent performance in work, and confidence in offering HBB were not significantly associated with the skills of health professionals on HBB. This is in line with the studies done in Ghana, Afghanistan, and Northwest and Northeast Ethiopia [24, 31, 42, 43]. But, incongruent with the study conducted in Eastern Ethiopia [32]. The reason for this may be methodological (having different sampling methods and procedures, study setting, and study period).

The main limitation of the study was that the study might be subjected to recall bias, the skills part was assessed by the interview that it may be their drawback. The causal association was under caution as the study design was cross-sectional. So, those limitations will be deciphered by assessing the skill part through direct observation on mannequins or if possible on real patients, and by doing follow up studies to develop cause and effect relationships.

The public health importance of this study was: The quality, and skilled care during prenatal, intrapartum, and postpartum periods are very pivotal for the health of the baby. The perinatal period is the most critical time in which most fetuses and newborns resulted in different complications. Hence, health professionals take the lion share in the health care system to avert those complications. The health professional’s skill on HBB is very essential to saving the life of the newborns those in need. Reduction in neonatal mortality is not that much satisfactory as compared to under 5 years of child deaths. The majority of neonatal deaths are related to intrapartum complications. One of the intrapartum complications which occurred commonly is birth asphyxia that could be managed by HBB. So, identifying the frequent barriers and giving immediate interventions is very essential.

This study showed the recent status of health professionals on the skills of HBB and the factors affecting it. This is very important and gives direction to do an intervention on the skills of HBB to improve the quality of intrapartum care and to reduce neonatal mortality.

In summary, there is a great controversy in previous studies to show the most frequent barriers that affect the skills of health care providers on HBB and rarity of information in Ethiopia that show the recent status of skills of HBB despite the improvement in neonatal health care services. Consequently, this study intended to fill those gaps. Training, well-equipped facility, and knowledge on HBB were identified as the most proximate, and modifiable factors for health professional’s skills on HBB as it was supported by different previous studies. Age was also another variable identified in this study and which is not modifiable. The readers should consider the limitations of this study while interpreting the finding, and the other scholars will do more to overcome those limitations. The finding of this study gives paramount importance for health care providers to identify there major gaps and barriers to the HBB. It is also very important for policymakers and program formulators to design different strategies for saving the lives of many newborns.

## Conclusions

This study indicated that health professionals’ good skills in HBB were optimum. Age, training on HBB, well-equipped facility, and adequate knowledge on HBB were identified as independent factors for health professional’s good skills on HBB. Interventions on strengthening strategies to address providing pre, in-service and refreshment training for health professionals on HBB and fulfill essential and priority items for health facilities that help to provide basic life support for the newborns. Different NGOs and stakeholders use this opportunity to contribute to the health care system to deliver training for health care providers and to well-equipped facilities to provide quality care. Educational institutions take the lion’s share to provide quality educations for future health professionals and to well simulate and training the students before going to health care institutions. Besides, it also gives support for health care institutions by providing refreshment pieces of training, short and long term capacity building activities. The future researchers ought to do this study by supplementing the qualitative part to dig out the unreachable facts with quantitative study and it is highly recommended to use prospective study designs for a better outcome.

## Supplementary information

**Additional file 1.** Questionnaire.

**Additional file 2.** STROBE checklist.

## Data Availability

The data will not be shared to preserve participant anonymity.

## Abbreviations

AHA: American Heart Association
HBB: Helping babies breathe
IESO: Integrated emergency surgery and obstetrics
NGO’s: Non-governmental organizations
NICU: Neonatal intensive care unit
OR: Operating room
