# Supplementary material for: Training and well-equipped facility increases the odds of skills of health professionals on helping babies breathe in public hospitals of Southern Ethiopia: cross-sectional study
Source: BMC Health Serv Res. 2019 Dec 9;19:946. doi: 10.1186/s12913-019-4772-z (PMC6902403; doi:10.1186/s12913-019-4772-z)
Supplement: Supplementary file 1 — Additional file 1. Questionnaire. [file 12913_2019_4772_MOESM1_ESM.pdf]

English Version Questionnaire

ARBA MINCH UNIVERSITY
COLLEGE OF MEDICINE AND HEALTH SCINCES
DEPARTMENT OF NURSING

Dear Respondents

This questionnaire is prepared to assess the skills of helping babies breathe and its associated factors among health professionals in hospitals of Southern Ethiopia, 2019. The questionnaire contains both closed and open ended questions and will be self-administered, some questions needs observation and interview of key informants. You are therefore kindly requested to provide genuine response to the questions. The information you provide is confidential and is used only for the purpose of this study. If you have any question, don‘t hesitate to ask the data collector.

Your cooperation and participation until the completion of the interview is very necessary for the successful completion of the assessment.

Thank you in advance for your cooperation!!!

Data collectors sign: _____________

Identification Number [ ]

S.No	Questions	Responses	Skip
**Part I: Socio- demographic characteristics**			
101	Age of the respondent	/_______/ in complete year	
102	Sex of respondent	1. Male<br>2. Female	
103	Marital status	1. Married<br>2. Divorced<br>3. Widowed<br>4. Single<br>5. Separated due to work	
104	Religion	1. Orthodox<br>2. Protestant<br>3. Catholic<br>4. Muslim<br>5. Traditional	
105	Salary/income	___________(in Ethiopian Birr)	
**Part II: Provider related questions**			
201	Type of professions?	1. Nurse<br>2. Midwives<br>3. Health officers<br>4. IESO<br>5. Medical doctors	
202	Qualification?	1. Diploma<br>2. BSc<br>3. MSc<br>4. General practitioner<br>5. Specialist(gynecologist, pediatrician)<br>6. Residents	
203	Type of facility they work?	1. Primary hospital<br>2. General hospital	
204	Geographic area/place of the hospital?	1. Zonal town<br>2. District town	
205	Type of ward they work?	1. Delivery<br>2. NICU<br>3. Pediatric ward<br>4. OR	
206	Year of experience in specified ward?	_____________(in complete month)	
207	Years of experience in work?	_____________(in complete years)	
208	Have you received any training in newborn resuscitation?	1. Yes<br>2. No →	Q#210
209	If yes, for above questions type of training received on newborn resuscitation?	1. Pre-service<br>2. In-service<br>3. Both pre and in service	
210	Do you have recent performance in offering HBB?	1. Yes<br>2. No	
211	Confidence in performing newborn resuscitation? ***(Observational; not provided)***	1. Very confident<br>2. Somewhat confident (needs coaching)<br>3. Not confident	
**Part III: Facility related checklists (to be observed and interviewed)**			

301	Number of deliveries in past 12 months	______________________	
302	Number of newborn deaths occurring within 24 hours after delivery in the past 12 months	________________________	
303	At least one newborn resuscitation performed in past 3 months	1. Yes<br>2. No	
304	Guidelines available in the ward?<br>***(Multiple response is possible)***	1. Management of newborn complications (NR)<br>2. Postpartum/postnatal care of newborns<br>3. Immediate newborn care	
305	Availability of essential items?<br>***(Multiple response is possible)***	1. Mucus extractor<br>2. Infant ambu bag<br>3. Infant face masks (sizes 0,1,2)<br>4. Towels or cloth for newborn<br>5. Newborn resuscitation table	
306	Availability of priority items?<br>***(Multiple response is possible)***	1. Syringes (1 ml, 2 ml, 5 ml, 10 ml)<br>2. Suction apparatus<br>3. Stethoscope for use with newborns<br>4. Source of warmth	
307	Does the facility have the mentioned infrastructure?<br><br>***(Multiple response is possible)***	1. Electricity<br>2. Generator<br>3. Availability of water in different parts of facility<br>4. Various kinds of telephone<br>5. Radio<br>6. Television<br>7. Light source<br>8. Ventilation<br>9. Toilet<br>10. Heating<br>11. Fan or air conditioning<br>12. Curtains for patient privacy<br>13. Waiting area	
**Part IV: Knowledge assessment tools**			
401	How would you diagnose birth asphyxia?<br>***(Multiple response is possible)***	1. Depressed breathing<br>2. Heart rate below 100 beats per minutes<br>3. Central cyanosis (blue tongue)	
402	What are the initial steps of newborn resuscitation?<br>***(Multiple response is possible)***	1. Place newborn face up<br>2. Wrap or cover baby<br>3. Position head so neck is slightly extended<br>4. Aspirate mouth and then nose<br>5. Explain to mother what is happening	
403	What do you do when resuscitating with a bag and mask or tube and mask?<br>***(Multiple response is possible)***	1. Place mask to cover chin, mouth and nose<br>2. Ensure seal between mask and face<br>3. Ventilate 1 or 2 times and see if chest is rising<br>4. Ventilate 40 times per minute for 1 minute<br>5. Pause to determine whether baby is breathing spontaneously	
404	What do you do if the baby is breathing and there is no sign of respiratory difficulty? After 30 seconds<br>***(Multiple response is possible)***	1. Keep baby warm<br>2. Initiate breastfeeding<br>3. Continue monitoring the baby	

405	What do you do if the baby does not begin breathing, breathing is less than 30 per minute, or if there is intercostal retraction or grunting?<br>***(Multiple response is possible)***	1. Continue to ventilate<br>2. Administer oxygen, if available<br>3. Assess the need for special care<br>4. Explain to mother what is happening	
**Part V: Skill assessment checklists (to be interviewed)**			
501	**Getting ready**<br>***(Multiple response is possible)***	1. Make sure equipment is ready for use<br>2. Wash hands and wear gloves<br>3. Quickly dry and wrap or cover the newborn<br>4. Place newborn on back on clean, warm surface<br>5. Tell woman what is going to be done, listen to her, and respond to her questions and concerns<br>6. Provide emotional support and reassurance	
502	**Resuscitation using bag and mask?**		
	Position head in slightly extended position?	1. Yes<br>2. No	
	Suction first the mouth and then the nose?	1. Yes<br>2. No	
	Introduce catheter into mouth and suction?	1. Yes<br>2. No	
	Introduce catheter into each nostril and suction?	1. Yes<br>2. No	
	Suction well if blood or meconium is in the newborn's mouth and/or nose?	1. Yes<br>2. No	
	If baby is still not breathing, start ventilating	1. Yes<br>2. No	
	Recheck position of newborn's head	1. Yes<br>2. No	
	Place correct-sized mask on newborn's face	1. Yes<br>2. No	
	Form a seal between mask and newborn's face	1. Yes<br>2. No	
	Squeeze bag	1. Yes<br>2. No	
	Check seal by ventilating and observing chest rise	1. Yes<br>2. No	
	If the newborn's chest IS rising:		
	Ventilate at 40 breaths/minute	1. Yes<br>2. No	
	Observe chest for easy rise and fall	1. Yes<br>2. No	
	If the newborn's chest IS NOT rising:		
	Check position of the head again	1. Yes<br>2. No	
	Reposition mask to improve seal	1. Yes<br>2. No	
	Squeeze the bag harder; repeat suction	1. Yes<br>2. No	
	Ventilate for 1 minute and then assess if the newborn is breathing spontaneously	1. Yes<br>2. No	

	If breathing is normal (no indrawing or grunting):		
	Place in skin-to-skin contact with mother	1. Yes<br>2. No	
	Observe breathing at frequent intervals	1. Yes<br>2. No	
	Encourage mother to begin breastfeeding	1. Yes<br>2. No	
	If newborn is breathing with severe indrawing:		
	Ventilate with oxygen, if available	1. Yes<br>2. No	
	Arrange immediate transfer for special care	1. Yes<br>2. No	
	If there is no gasping or breathing at all after 20 minutes of ventilation, stop ventilating	1. Yes<br>2. No	
	**Post-procedure tasks**		
	Place disposable suction catheters and mucus extractors in leak-proof container	1. Yes<br>2. No	
	For reusable catheters and mucus extractors:		
	Place in chlorine solution for 10 minutes	1. Yes<br>2. No	
	Wash in water and detergent	1. Yes<br>2. No	
	Use a syringe to flush catheters/tubing	1. Yes<br>2. No	
	Boil or disinfect in chemical solution	1. Yes<br>2. No	
	Take apart valve/mask and inspect for cracks/tears	1. Yes<br>2. No	
	Wash valve/mask and check for damage	1. Yes<br>2. No	
	Select sterilization or high-level disinfection method	1. Yes<br>2. No	
	Wash hands and dry with clean cloth or air dry	1. Yes<br>2. No	
	After chemical disinfection, rinse all parts with clean water and allow to air dry	1. Yes<br>2. No	

Thank you for your cooperation!!!
