# Supplementary material for: Training and well-equipped facility increases the odds of skills of health professionals on helping babies breathe in public hospitals of Southern Ethiopia: cross-sectional study
Source: BMC Health Serv Res. 2019 Dec 9;19:946. doi: 10.1186/s12913-019-4772-z (PMC6902403; doi:10.1186/s12913-019-4772-z)
Supplement: Supplementary file 2 — Additional file 2. STROBE checklist. [file 12913_2019_4772_MOESM2_ESM.pdf]

STROBE Statement—Checklist of items that should be included in reports of ***cross-sectional studies***

	**Item No**	**Recommendation**
**Title and abstract**	1	(*a*)Training and Well-equipped Facility Increases the Odds of Skills of Health Professionals on Helping Babies Breathe in Public Hospitals of Southern Ethiopia: **Cross-sectional Study**
		(*b*) **Abstract**<br><br>**Background:** Good skills of health professionals on helping babies breathe is essential for improving the neonatal outcome. There is great controversy between studies to show the most proximate factors of skills of health care providers about helping babies breathe. There was also paucity of information's in Ethiopia that show the recent status of skills about helping babies breathe despite the improvement in neonatal health care services. Therefore, this study intends to fill those gaps in assessing skills of helping babies breathe and its associated factors among health professionals in public hospitals of Southern Ethiopia.<br><br>**Methods:** A facility-based cross-sectional study design was conducted among 441 health professions from March 10-30, 2019. A simple random sampling method was used to select study participants. Pre-tested interviewer-administered questionnaire and observational checklist was used to collect the data. Data were entered in to Epi data version 3.1 and exported to SPSS version 25 for analysis. Bivariate and multivariable analysis was done by using binary logistic regression. In this study P-value < 0.05 was considered to declare factors as statistically significant.<br><br>**Results:** In this study 71.1% (95%CI: 66.2%, 75.4%) of health professionals had good skills on helping babies breathe. Age group from 25-34 (AOR=2.24; 95%CI: 1.04, 4.81), training (AOR=2.69; 95%CI: 1.49, 4.87), well-equipped facility (AOR=2.15; 95%CI: 1.09, 4.25) and adequate knowledge on helping babies breathe (AOR=2.21; 95%CI: 1.25, 3.89) were significantly associated with health professionals good skills on helping babies breathe.<br><br>**Conclusions:** This study point out that good skills of health professionals on helping babies breathe was optimum. For better improvement health care providers should be frequently trained and the health facilities should be equipped with adequate materials to provide basic life support for the newborns in need.<br><br>**Keywords:** Helping Babies Breathe, Neonatal Resuscitation, and Management of Neonatal Complications
**Introduction**		
Background/rationale	2	**Background**<br><br>Achieving the Sustainable Development Goal (SDG) targets for ending preventable mortality and provision of universal health coverage will require large-scale approaches to improving quality of health care [1]. There is a growing interest for the need to strengthen quality of care globally [2]. Remarkable progress has been made in recent decades to reduce the number of child deaths worldwide, but neonatal mortality rates have declined at a slower pace [3].<br><br>Globally, neonatal mortality accounts for an increasing proportion of mortality in children aged <5 years. Most newborn deaths occur in low- and middle-income countries. Two-thirds of all newborn mortality is found in 12 countries, six of which are in Sub-Saharan Africa [4, 5]. Birth asphyxia, or failure to initiate and sustain spontaneous breathing at birth, contributes to approximately 27% to 30% of neonatal deaths in resource-limited countries [6]. An estimated two-thirds of the world's 2.7 million newborn deaths may be prevented with basic quality care at birth and during the postnatal period [7, 8].<br><br>On a global scale, many successes have been achieved in the implementation of helping babies breathe (HBB) [8]. Immediately after birth, infants who are breathing and crying may undergo delayed cord clamping. However, until more evidence is available, infants who are not breathing or crying should have the cord clamped, so that resuscitation measures can commence promptly [9]. Helping babies breathe is a set of interventions used

to assist the airway, breathing and circulation of a newborn following birth or to help it breathe and to help its heart beat [10, 11]. Infants who required face mask ventilation (FMV) were more likely to die particularly when the intervention was delayed or prolonged [12].

Health care providers are the key personnel in the management of HBB. The science of resuscitation demonstrates that more than 95% of babies will respond to simple steps of drying, stimulation, warmth, suctioning if needed, and bag-mask ventilation [13, 14]. Effective application of neonatal resuscitation for those in need are identified substantial missed opportunities to provide lifesaving care and highlighted the need to improve the availability of essential equipment and ensure that birth attendants acquire and maintain their skills to provide high quality newborn care[15]. Availability and functionality of necessary equipment and trained staff to use the equipment will be important to realize the potential gains that can be achieved through provision of HBB [7, 8]. Good skill of health professionals on HBB is essential for improving the neonatal outcome. It plays a major role in early diagnosis, appropriate management and, accordingly, reduction of adverse consequences [16, 17]. Health workers have the power to change health care routines in daily practice, but this must be accompanied by concrete inputs at every level of the health system [18].

Success of HBB is depends on provider's clinical skill as well as how to perform essential steps and how to access basic HBB equipment's. Report from studies done in Africa and Asian countries showed that well trained health professionals on HBB are not steadily available in all health care facilities [19, 20]. Studies conducted in Tanzania stated that 32.4% health care providers had adequate skills about helping babies breathe. Despite, health worker knowledge using a case study significantly improved as well, from 23% to 41% but skills in resuscitation using a newborn mannequin were persistently low [21, 22]. Similarly, a study in Cameron showed health care providers were competent at providing essential newborn care but they lacked skills for proper handling of newborns that do not breathe at birth. The score was 24% at neonatal resuscitation tasks [23]. In Nigeria, only 3% of 117 birth attendants interviewed demonstrated competence to undertake resuscitation, all of whom were classified as skilled attendants and worked in referral facilities [15]. The overall skills of midwives, nurses, pediatrics residents and obs-gyn residents were insufficient as shown on a study done in Ethiopia. The overall mean skill scores of health professionals (midwives, nurses and residents) were 6.8 (SD=3.9) [24].

Lack of professional support and infrequent resuscitation skills practice are commonly cited as barriers to skill retention after HBB training [25]. Working facility, experience, and equipment was significant predictor of skills [22]. Other studies from low- and middle-income countries have shown similar barriers to newborn resuscitation, including limited provider knowledge and skills, inadequate training, and poor availability of equipment [13, 23, 26, 27].

Skills learned in standardized courses are estimated to last only a few months. Neonatal Resuscitation Program skills or HBB deteriorate immediately after certification. Significant skill deficits were seen at baseline raising concerns regarding the efficacy of the current course structure. Discrepancies in knowledge and skill retention may impact caregiver performance [28]. The low performance and lack of improvement in newborn resuscitation raises the question of what needs to be done to improve providers' skills in newborn resuscitation, especially at lower-level health facilities with low delivery report, where providers may not have the occasion to perform resuscitation frequently [21].

There is great controversy between studies to show the significant factors that affect skills of health care providers. Some studies did not assess the most proximal factors for skills on HBB like facility related factors. But, others studies showed insignificant association and does not clearly assessed skills of health care providers as well as their association. In addition, there is paucity of information's in Ethiopia that show the skills about HBB despite the improvement in neonatal health care services to the knowledge of investigator. Therefore, this study fills those gaps by assessing the current skills of HBB and factors affecting in hospitals of Gamo, Gofa, Segen Areas People, Konso and South Omo Zone, Southern Ethiopia.

Objectives	3	To assess the skills of health professionals on helping babies breathe in hospitals of Gamo, Gofa, Segen Areas People, Konso and South Omo Zone, Southern Ethiopia.

		To identify factors associated with skills of health professionals on helping babies breathe in hospitals of Gamo, Gofa, Segen Areas People, Konso and South Omo Zone, Southern Ethiopia.
**Methods**		
Study design	4	A facility-based cross-sectional study design was employed.
Setting	5	This study was conducted in Hospitals of Gamo, Gofa, Segen Areas People, Konso and South Omo Zone, Ethiopia from March 10-30, 2019. Gamo, Gofa, Segen Areas People, Konso and South Omo area are administrative Zones in Southern part of Ethiopia. Those Zones hosted different general and primary hospitals which serve the community by providing preventive and curative services. There are five functional hospitals in Gamo Zone (Arba Minch General Hospital, Chencha Primary Hospital, Kamba Primary Hospital, Gerese Primary Hospital and Selamber Primary Hospital), one hospital in Gofa Zone (Sawla General Hospital), two hospitals in Segen Areas People Zone (Gidole Primary Hospital and Amaro Kele Primary Hospital), one hospital in Konso Zone (Karat Primary Hospital) and two hospitals in South Omo Zone (Jinka General Hospital and Gazer Primary Hospital).<br><br>The data were collected by using pre-tested structured both self and interview administered questionnaires and observational checklist. Some of the tools were developed or adapted from different literatures and guidelines. But, to measure the outcome variables standard questionnaire was used which were developed, reviewed, revised and approved in different workshops including the Ministry of Public Health (MoPH), national EmONC trainers, UNICEF, the World Health Organization, and experts from nongovernmental organizations (NGOs) contracted by the MoPH to operate primary health facilities offering the Basic Package of Health Services [22, 29-32]. The tool contains five main parts: basic characteristics, provider related questions, facility assessment tools (observational check list and interview), knowledge assessment tools and skill assessment tools. Pre-test was carried out on 5% of study subjects in other health care institution and necessary modifications and amendments were taken accordingly before actual data collection.<br><br>Data were collected by well-trained three BSc holder nurses and six midwives and the collection process were supervised by three trained supervisors. The data were collected from health professions in nine selected hospitals which were found in five zones of Southern Ethiopia. The data collectors were give information about the study aim and the possible procedures for study participants before providing the questionnaire and interviewing the study participants. After stated the study aim, the data collectors offered self-administered questionnaire to assess the knowledge of helping babies breathe. Then, each study participants were interviewed for skills of helping babies breathe in room which was arranged by data collectors during off work time after returned the self-administered questionnaire. At the end, the hospitals were assessed for availability of guidelines, essential items and infrastructures by interviewing key informants and observation by using checklists.
Participants	6	(*a*)<br>All health professionals who were staff in respective wards in each hospital were included in this study whereas those health professionals on annual leave at the time of data collection were excluded from this study.<br><br>The source population for this study was all health professionals who were working in Hospitals of Gamo, Gofa, Segen Areas People, Konso and South Omo Zone, Southern Ethiopia.<br><br>The study population for this study was all health professionals who were working in delivery, neonatal intensive care unit (NICU), pediatric wards and operating room (OR) in selected Hospitals.<br><br>Those selected health professions from respective wards and hospitals and satisfying the inclusion criteria were the sampled population.<br><br>There are eleven fully functional hospitals in five Zones of Southern Ethiopia (Gamo, Gofa, Segen Areas People, Konso and South Omo Zone). From them, nine hospitals were selected by simple random sampling method. Initially, the calculated sample size was proportionally

		allocated to each hospital based on the number of health professionals who were working in the respective wards of each hospital. Secondly, based on proportions each health professionals were selected by using simple random sampling method after generating a table of the random number to come up with the calculated sample size.
Variables	7	The dependent variable for this study was skills on HBB, and basic or socio-demographic characteristics, provider-related factors, facility-related factors, and knowledge on HBB were independent variables.
Data sources/ measurement	8*	**Measurements**<br>Table 1: Operational definitions of variables and measurements for study conducted among health professionals in public hospitals of Southern Ethiopia, 2019

Variables	Descriptions
Health professionals	Health care providers (nurse, midwives, health officers, Integrated Emergency Surgery and Obstetrics (IESO) and medical doctors) who were working in the delivery ward, NICU, pediatric ward and OR and have access to HBB.
Knowledge of health professionals on HBB	Level of awareness of the health care providers on HBB. Knowledge of health professionals was considered adequate when they answered correctly at least 16 out of 20 knowledge assessing questions on helping babies breathe (total score of ≥80%) after following algorithms for the main American Heart Association (AHA) advanced cardiac life support, and inadequate when they correctly answered less than 16 out of 20 questions (total score <80%) [32, 46].
Skills of health professionals on HBB	Skills of health professionals were good for who responded correctly at least 32 of the 40 activities on skill assessment tools for HBB (total score of ≥80%) after following algorithms for the main AHA advanced cardiac life support, and poor for who correctly responded for less than 32 of the 40 activities (total score <80%) [24, 31].
Well-equipped facility	Those facilities that have both essential items (mucus extractors, infant ambu bag, face masks, towels, and newborn resuscitation table) and priority items (syringes, suction apparatus, stethoscope for use with newborns and source of warmth).

Bias	9	Maximum efforts was done to decrease recall bias by probing and dig outing the points.
Study size	10	The calculated sample size was 441. But, finally 429 study participated were involved, yielding a response rate of 97.3%.
Quantitative variables	11	Summery statistics (mean and SD), and recoding was done
Statistical methods	12	Descriptive analysis was done by computing proportions and summary statistics. Then the information was presented by using simple frequencies, summary measures, tables and figures. Bi-variate analysis was used to see the association between each independent variable and the outcome variables by using binary logistic regressions. The assumptions for binary logistic regression were checked. The goodness of fit was tested by Hosmer-Lemeshow statistic and Omnibus tests. All variables with P<0.25 in the bivariate analysis were included in the final model of multivariable analysis in order to control all possible confounders. In addition, variables which were significant in previous studies and from context point of view included in the final model even if the above criteria will not meet. Multicollinearity test was carried out to see the correlation between independent variables by using collinearity statistics (Variance inflation factor (VIF) >10 and tolerance (T) <0.1 were considered as suggestive of existence of multi co-linearity). The strength of statistical association was measured by Odds Ratio with 95 % CI. Adjusted Odds Ratio along with 95% CI was estimated to identify associated factors for skills of HBB by using binary logistic regressions in multivariable analysis. In this study P-value < 0.05 was considered to declare a result as statistically significant.<br>In general, we haven't encountered missing data.

Results

Participants	13*	(a) 429 health professionals were involved from 9 hospitals of southern Ethiopia which gives response rate of 97.3%.
		(b) The mean reason for non-participation were unwillingness.
		(c) For the diagram; (See figure 1 from main manuscript)
Descriptive data	14*	(a)

Socio-demographic characteristics of the respondents

In this study, 429 health professionals participated, yielding a response rate of 97.3%. The mean age and standard deviation of study participants were 29.7±SD4.88 years old. More than half (55.9%) of the study participants were females and 281(65.5%) were married. Two hundred (46.6%) of health professionals were orthodox religion follower (Table 2).

Table 2: Socio-demographic characteristics of the health professionals in hospitals of Southern Ethiopia, 2019

Characteristics	Frequency	Percentage (%)
**Age of respondent**		
15-24	48	11.2
25-34	317	73.9
≥35	64	14.9
**Sex**		
Male	189	44.1
Female	240	55.9
**Marital status**		
Married	281	65.5
Divorced	17	4
Widowed	4	0.9
Single	127	29.6
**Religion**		
Orthodox	200	46.6
Protestant	207	48.3
Catholic	6	1.4
Muslim	16	3.7
**Salary**		
<3579ETB	68	15.9
3579-5452ETB	254	59.2
>5452ETB	107	24.9

Note: *1ETB=0.036USD*

Provider related factors

Of the total respondents, 248(57.8%) were nurses and 213(49.7%) were diploma holder. Two hundred forty-three (56.6%) of health professionals were working in general hospitals. One hundred sixty-seven (38.9%) of the health professionals had work experience of 3 to 6 years and almost half (52.0%) had been working for 12 to 52 month the in the specified ward. Out of the total study participants, 333(77.6%) received training on HBB and 375(87.4%) had recent involvement in the help to baby breath. Regarding the type of the training, 135(40.6%), 117(35.1%) and 81(24.3%) received pre, in and both pre and in-service respectively (Table 3).

Table 3: Provider related factors of the health professionals in hospitals of Southern Ethiopia, 2019

Variables	Frequency	Percentage (%)
**Profession**		
Nurse	248	57.8
Midwives	117	27.3
Health Officers	28	6.5
IESO	28	6.5
Medical Doctors	8	1.9
**Qualification**		
Diploma	213	49.7
BSc	173	40.3
MSc	35	8.2

General Practitioner	6	1.4
Specialists®	2	0.5
**Unit of service**		
Delivery	146	34.0
NICU	92	21.4
Pediatric Ward	130	30.3
OR	61	14.2
**Year of experience in specified ward**		
<12 month	112	26.1
12-52 month	223	52.0
>52 month	94	21.0
**Year of experience in work**		
<3 year	172	40.1
3-6 year	167	38.9
>6 year	90	21.0
**Training on HBB**		
Yes	333	77.6
No	96	22.4
**Recent involvement in HBB**		
Yes	375	87.4
No	54	12.6

®obstetrician and gynecologist, pediatrician

Facility related factors

The number of deliveries occurred in facilities of Gamo, Gofa, Segen areas people, Konso and South Omo Zone in the past 12 months was ranged from 487 to 4640 and on average 1488 deliveries. On the other hand, the number of newborn deaths occurred within 24 hours after delivered in the past 12 months was ranging from 3 to 46 and 11 deaths on average. All the facilities were assessed for the performance of newborn resuscitation and all had performed to the minimum of one newborns resuscitation in the past 3 months. In addition, facilities were categorized as a primary and general hospital and assessed for the availability of guidelines, essential items, priority items and infrastructures (Table 4).

Table 4: Facility related factors for the study conducted among health professionals in public hospitals of Southern Ethiopia, 2019

**Facility**	**Primary Hospitals(n=6)**		**General Hospitals (n=3)**	
	**Frequency**	**(%)**	**Frequency**	**(%)**
**Availability of guidelines**				
Management of newborn complications	6	100	3	100
Postnatal care of newborns	4	66.7	2	66.7
Immediate newborn care	6	100	2	66.7
**Availability of essential items**				
Mucus extractor	6	100	2	66.7
Infant ambu bag	6	100	2	66.7
Infant face masks (sizes 0,1,2)	6	100	3	100
Towels or cloth for newborn	6	100	2	66.7
Newborn resuscitation table	6	100	3	100
**Availability of priority items**				
Syringes (1 ml, 2 ml, 5 ml, 10 ml)	6	100	2	66.7
Suction apparatus	6	100	3	100
Stethoscope for use with newborns	4	66.7	3	100
Source of warmth	6	100	3	100
**Infrastructures*(mean ± SD)**	50(1.17)		33.3(1.53)	

**electricity, generator, availability of water in different parts of the facility, various kinds of telephone, radio, television, light source,*
ventilation, toilet, heating, fan or air conditioning, curtains for patient privacy and waiting

Knowledge of helping babies breathe

Regarding diagnosis of birth asphyxia, 390(90.9%) stated depressed breathing and 356(83.0%) indicated initial steps of newborn resuscitation were positioning the head to slightly extended neck. Four hundred fifteen (96.7%) reported place mask to cover chin, mouth, and nose while resuscitating with a bag and mask or tube and mask. Of the total respondents, 384(89.5%) stated initiate breastfeeding after 30 seconds if the baby is breathing and there is no sign of respiratory difficulty. The majority (90.9%) of the study participants stated that administer oxygen, if available and 370(86.2%) reported continue to ventilate if the baby does not begin breathing, or breathing is less than 30 per minute, or if there is an intercostal retraction or grunting (Table 5).

Table 5: Knowledge of helping babies breathe among health professionals in public hospitals of Southern Ethiopia, 2019

Categories	Frequency	Percentage (%)
**How do you diagnose birth asphyxia?**		
Depressed breathing	390	90.9
HR<100/min	354	82.5
Central cyanosis	340	79.3
**What are the initial steps of newborn resuscitation?**		
Place newborn face up	343	80.0
Wrap or cover baby	355	82.8
Position head so the neck is slightly extended	356	83.0
Aspirate mouth and then nose	342	79.7
Explain to mother what is happing	344	80.2
**What do you do when resuscitating with a bag and mask or tube and mask?**		
Place mask to cover chin, mouth, and nose	415	96.7
Ensure seal between mask and face	387	90.2
Ventilate 1 or 2 times and see if the chest is rising	386	90.0
Ventilate 40 times per minute for 1 minute	359	83.7
Pause to determine whether the baby is breathing spontaneously	356	83.0
**What do you do if the baby is breathing and there is no sign of respiratory difficulty? After 30 seconds**		
Keep baby warm	383	89.3
Initiate breastfeeding	384	89.5
Continue monitoring the baby	379	88.3
**What do you do if the baby does not begin breathing, breathing is less than 30 per minute, or if there is intercostal retraction or grunting?**		
Continue to ventilate	370	86.2
Administer oxygen, if available	390	90.9
Assess the need for special care	350	81.6
Explain to mother what is happening	350	81.6

Overall, 76.2% (95%CI: 72.2%, 80.3%) of the study participants had adequate knowledge on helping babies breathe.

(b) No missing data

Outcome data 15*

Skills of helping babies breathe

Of the study participants, 347(80.9%) itemized make sure equipment is ready for use before staring resuscitating with bag and mask and 404(94.2%) stated position head in a slightly extended position while resuscitating by using bag and mask. During ventilation, three hundred forty-eight (81.1%) stated that observe chest for easy rise and fall if the newborns chest is not rising 376(87.6%) reported that reposition mask to improve seal. After resuscitation, if breathing is normal (no indrawing or grunting) 386(90.0%) of study participants stated place the baby in skin-to-skin contact with mother and observe breathing at frequent intervals. The majority (90.0%) stated that if newborn breaths with severe chest indrawing, ventilate with oxygen if available. Three hundred fifty-five (82.8%) stated that disinfect mucus extractors with chemical, and rinse all parts with clean water and allow to air dry for reusable (Table 6).

Table 6: Skills of helping babies breathe among health professionals in public hospitals of Southern Ethiopia, 2019

Categories	Frequency	Percentage (%)
**Getting ready**		
Make sure equipment is ready for use	347	80.9
Wash hands and wear gloves	343	80.0
Quickly dry and wrap or cover the newborn	314	73.2
Place newborn on back on clean, warm surface	288	67.1
Tell women what is going to be done, listen to her, and respond to her questions and concerns	282	65.7
Provide emotional support and reassurance	282	65.7
**Resuscitating using bag and mask?**		
Position head in slightly extended position	404	94.2
Suction first the mouth and then the nose	392	91.4
Introduce catheter in to mouth and suction	372	86.7
Introduce catheter in to each nostril and suction	376	87.6
Suction well if blood or meconium is on the newborn’s mouth and/or nose	383	89.3
If baby is still not breathing, start ventilating	390	90.9
Recheck position of newborn’s head	373	86.9
Place correct-sized mask on newborn’s face	367	85.5
Form a seal between mask and newborn’s face	350	81.6
Squeeze bag	338	78.8
Check seal by ventilating and observing chest rise	353	82.3
**If the newborn’s chest is rising:**		
Ventilate at 40 breaths/minute	344	80.2
Observe chest for easy rise and fall	348	81.1

**If the newborn's chest is not rising:**		
Check position of the head again	367	85.5
Reposition mask to improve seal	376	87.6
Squeeze the bag harder; repeat suction	361	84.1
Ventilate for 1 minute and then assess if the newborn is breathing	363	84.6
**If breathing is normal (no indrawing or grunting):**		
Place in skin-to-skin contact with mother	386	90.0
Observe breathing at frequent intervals	386	90.0
Encourage mother to begin breastfeeding	378	88.1
**If newborn is breathing with severe indrawing:**		
Ventilate with oxygen, if available	386	90.0
Arrange immediate transfer for special care	354	82.5
If there is no gasping or breathing at all after 20 minutes of ventilation, stop ventilating	352	82.1
**Post-procedure tasks**		
Place disposable suction catheters and mucus extractors in leak-proof container	354	82.5
**For reusable catheters and mucus extractors**		
Place in chlorine solution for 10 minutes	354	82.5
Wash in water and detergent	354	82.5
Use a syringe to flush catheters/tubing	351	81.8
Boil or disinfect in chemical solution	334	77.9
Take apart valve/mask and inspect for cracks/tears	333	77.6
Wash valve/mask and check for damage	339	79.0
Select sterilization or high-level disinfection method	346	80.7
Wash hands and dry with clean cloth or air entry	350	81.6
After chemical disinfection, rinse all parts with clean water and allow to air dry	355	82.8

Overall, 71.1% (95%CI: 66.8%, 75.4%) of the study participants had good skills on helping babies breathe.

Main results 16

(*a*)

Factors associated with skills of helping babies breathe

After controlling for confounders in multivariable model age, training on HBB, well-equipped facility and adequate knowledge on HBB had a significantly associated with health professional's skills of HBB.

Health professionals whose age ranged from 25-34 years old were 2.24 times more likely had skills on HBB as compared to age group from 15-24 years old (AOR=2.24,95%CI:1.04, 4.81). Those who received training on HBB were 69% more likely had skills on HBB (AOR=2.69, 95%CI: 1.49, 4.87). The odds of skills of HBB among health professionals who working in well-equipped facility were 2.15 times (AOR=2.15, 95%CI: 1.09, 4.25). Those health care providers who had adequate knowledgeable on HBB were 2.21 times more likely had skills on HBB (AOR=2.21, 95%CI: 1.25, 3.89) (See table 7).

Table 7: Factors associated with skills of helping babies breathe among health professionals in public hospitals of Southern Ethiopia, 2019

**Variables**	**Skills of HBB**		**Crude OR**	**Adjusted OR**
	**Good**	**Poor**	**95%CI**	
**Age**				
15-24	23(47.9%)	25(52.1%)	1	1
25-34	236(74.4%)	81(25.6%)	3.17(1.70,5.89)	2.24(1.04,4.81)*
>34	46(71.9%)	18(28.1%)	2.78(1.27,6.09)	1.92(0.68,5.39)
**Salary**				
<3579ETB	42(61.8%)	26(38.2%)	1	1
3579-5452ETB	174(68.5%)	80(31.5%)	1.35(0.77,2.35)	0.90(0.43,1.89)
>5452ETB	89(83.2%)	18(16.8%)	3.06(1.51,6.19)	1.13(0.38,3.42)
**Profession**				
Nurse	167(67.3%)	81(32.7%)	0.64(0.42,0.99)	0.72(0.43,1.22)
Other©	138(76.2%)	43(23.8%)	1	1
**Qualification**				
Diploma	133(62.4%)	80(37.6%)	0.43(0.28,0.66)	0.55(0.29,1.03)
Other®	172(79.6%)	44(20.4%)	1	1
**Year of experience in specified ward**				
<12 month	60(53.6%)	52(46.4%)	1	1
12-52 month	169(75.8%)	54(24.2%)	2.71(1.68,4.39)	1.61(0.90,2.86)
>52 month	76(80.9%)	18(19.1%)	3.66(1.94,6.89)	1.83(0.83,4.03)
**Year of experience in work**				
<3 year	116(67.4%)	56(32.6%)	1	1
3-6 year	120(71.9%)	47(28.1%)	1.23(0.78,1.96)	0.99(0.54,1.82)
>6 year	69(76.7%)	21(23.3%)	1.59(0.89,2.84)	0.90(0.41,1.97)
**Training on HBB**				
Yes	258(77.5%)	75(22.5%)	3.59(2.23,5.77)	2.69(1.49,4.87)*
No	47(49.0%)	49(51.0%)	1	1
**Recent performance on offering HBB**				

Yes	281(74.9%)	94(25.1%)	3.74(2.08,6.71)	1.78(0.82,3.89)
No	24(44.4%)	30(55.6%)	1	1
**Confidence on offering HBB**				
Very confident	237(74.8%)	80(25.2%)	3.56(1.71,7.38)	1.68(0.67,4.23)
Somewhat confide.	53(67.1%)	26(32.9%	2.45(1.07,5.61)	1.99(0.72,5.50)
Not confident	15(45.5%)	18(54.5%)	1	1
**Well-equipped facility**				
Yes	277(75.3%)	91(24.7%)	3.59(2.06,6.26)	2.15(1.09,4.25)*
No	28(45.9%)	33(54.1%)	1	1
**Knowledge on HBB**				
Inadequate	47(46.1%)	55(53.9%)	1	1
Adequate	258(78.9%)	69(21.1%)	4.38(2.73,7.01)	2.21(1.25,3.89)*

©Midwives, Health officers, IESO and medical doctors, ® BSc, MSc, GP and Specialists and **Significant at P<0.05*

Other analyses	17	N/A
**Discussion**		
Key results	18	In this study 71.1% (95%CI: 66.8%, 75.4%) of the study participants had good skills on HBB. Age, training on HBB, well-equipped facility, and knowledge on HBB had significantly associated with health professionals skills on HBB.<br><br>The magnitude of good skills of health professionals on HBB were in line with study done in Afghanistan (66% and 71%) and higher than studies done in Tanzania (32.4%), Iraq (52%) and Ethiopia (55.8%) [22, 24, 31, 33]. The reason for this discrepancy on skills of HBB are due to the fact that there is advance in the health care system and refreshment trainings are given for health professionals by concerning bodies.<br><br>Age of the respondents ranged from 25-34 years old was significantly associated with skills of HBB. But, this was incongruent with study done in Ethiopia [24]. This is difference may be due methodological, those health professionals whose age was in this category where received training on HBB, and majority of health professionals are in this age group as from normal demography. Finding from this study showed that training on HBB, and working in well-equipped facilities were significantly associated with health professional's skills on HBB. This is in line with different studies done Kenya, Afghanistan, Rwanda, Tanzania, India, developing countries, Ethiopia, Nepal and Global Network research sites (Nagpur and Belgaum, India and Eldoret, Kenya), low income countries, resource limited settings, Cameroon and Dominican Republic [4, 13, 17, 22-24, 26, 32, 34-40]. The reason this is due to the fact health professionals who received training as well as refreshment training has recent memory and actively performs the activities. Similarly, those providers who are working in well-equipped facilities is frequent exposure with cases and equipment that used for HBB and developing their own skills from time to time.<br><br>As revealed in study knowledge of health professionals on HBB were significantly associated with skills of HBB. This is consistent with studies conducted in Kenya, Cameroon, Nepal and Ethiopia [13, 23, 26, 27]. The fact for this is knowledgeable health care professionals are more likely having skills and perform the activities in well manner as much as possible.
Limitations	19	The main limitation of the study was that the study might be subjected to recall bias, the skills part was assessed by interview that it may be their own drawback. The causal association was under caution as the study design was cross-sectional.
Interpretation	20	The results were interpreted with cautions as per recall bias and limitations of the study
Generalisability	21	This study was generalizable to source as per the sample size adequate and response rate was high.
**Other information**		
Funding	22	Arba Minch University provided funds for the data collection and stationary materials of this research work with a project grant code of Acct. No GOV-1000021708902. The website of the university is www.amu.edu.et. "The funders had no role in study design, data collection and analysis, decision to publish, or preparation of the manuscript."
